# PERCEPTIONS OF DISCHARGE READINESS AND ENGAGEMENT IN DISCHARGE PLANNING FOR SPOUSAL VERSUS NONSPOUSAL CAREGIVERS

**DOI:** 10.1093/geroni/igac059.909

**Published:** 2022-12-20

**Authors:** Catherine Elmore, Alycia Bristol, Lisa Barry, Eli Iacob, Erin Johnson, Andrea Wallace

**Affiliations:** University of Utah, Salt Lake City, Utah, United States; University of Utah, Salt Lake City, Utah, United States; University of Utah, Salt Lake City, Utah, United States; University of Utah, Salt Lake City, Utah, United States; University of Utah, Salt Lake City, Utah, United States; University of Utah, Salt Lake City, Utah, United States

## Abstract

Informal caregivers are frequently excluded during hospital discharge planning, potentially impacting their ability to effectively care for older adults at home. Few studies have examined experiences of spousal versus non-spousal caregivers during hospital discharge planning. In a secondary analysis of a mixed-method study, we quantitatively examined how spousal relationships impact caregivers’ (n=266; 51.8% identified as a spouse or partner) scores of patient discharge readiness using the Readiness for Hospital Discharge Scale (RHDS-CG). We then conducted semi-structured interviews with a participant subset (n=23), and analyzed transcribed interviews using content analysis. First, comparing scores on the RHDS-CG, spouses/partners (88.4%) were more likely than non-spouses (75%) to report RHDS scores of 7+ corresponding with moderate to high readiness (X2 (1) = 8.070, p=.005). Among those interviewed, spouses/partners (65.2%) described their role as long-term, and shared strategies they had learned over time regarding how to seek involvement with healthcare professionals (HCPs). In contrast, non-spousal caregivers (34.8%) viewed their role as short term and struggled with how to communicate with HCPs, citing patient privacy rules and patient autonomy as perceived barriers. Overall, spousal caregivers had more experience with the healthcare system and felt better prepared to assume post-discharge care duties. Exploring the experiences of non-spousal caregivers, which make up more than one-third of our sample, is important since caregiving roles shift away from spouses to adult children and others as people age. Further consideration is necessary regarding how to support non-spousal caregivers in navigating the healthcare system.

